# The DrugAge database of aging‐related drugs

**DOI:** 10.1111/acel.12585

**Published:** 2017-03-16

**Authors:** Diogo Barardo, Daniel Thornton, Harikrishnan Thoppil, Michael Walsh, Samim Sharifi, Susana Ferreira, Andreja Anžič, Maria Fernandes, Patrick Monteiro, Tjaša Grum, Rui Cordeiro, Evandro Araújo De‐Souza, Arie Budovsky, Natali Araujo, Jan Gruber, Michael Petrascheck, Vadim E. Fraifeld, Alexander Zhavoronkov, Alexey Moskalev, João Pedro de Magalhães

**Affiliations:** ^1^ Integrative Genomics of Ageing GroupInstitute of Ageing and Chronic DiseaseUniversity of LiverpoolLiverpoolUK; ^2^ Amrita School of BiotechnologyAmrita Vishwa Vidyapeetham (Amrita University)CoimbatoreIndia; ^3^ Energy Metabolism LaboratorySwiss Federal Institute of Technology (ETH) ZurichZurichSwitzerland; ^4^ Department of BiophysicsFederal University of São PauloSão PauloBrazil; ^5^ The Shraga Segal Department of Microbiology, Immunology and GeneticsCenter for Multidisciplinary Research on AgingBen‐Gurion University of the NegevBeer ShevaIsrael; ^6^ Judea Regional Research & Development CenterCarmel90404Israel; ^7^ Department of ScienceYale‐ NUS CollegeSingapore City138527Singapore; ^8^ Department of BiochemistryYong Loo Lin School of MedicineNational University of SingaporeSingapore City117597Singapore; ^9^ Department of Chemical PhysiologyThe Scripps Research InstituteLa JollaCAUSA; ^10^ Pharmaceutical Artificial Intelligence Research DivisionEmerging Technology CentersInsilico Medicine, IncJohns Hopkins University at EasternB301, 1101 33rd StreetBaltimoreMD21218USA; ^11^ The Biogerontology Research FoundationOxfordUK; ^12^ Moscow Institute of Physics and TechnologyDolgoprudny141700Russia; ^13^ Laboratory of Molecular Radiobiology and GerontologyInstitute of Biology of Komi Science Center of Ural Branch of Russian Academy of SciencesSyktyvkar167982Russia; ^14^ Engelhardt Institute of Molecular Biology of Russian Academy of SciencesMoscow119991Russia

**Keywords:** bioinformatics, compound, functional genomics, lifespan, longevity, pharmacology

## Abstract

Aging is a major worldwide medical challenge. Not surprisingly, identifying drugs and compounds that extend lifespan in model organisms is a growing research area. Here, we present DrugAge (http://genomics.senescence.info/drugs/), a curated database of lifespan‐extending drugs and compounds. At the time of writing, DrugAge contains 1316 entries featuring 418 different compounds from studies across 27 model organisms, including worms, flies, yeast and mice. Data were manually curated from 324 publications. Using drug–gene interaction data, we also performed a functional enrichment analysis of targets of lifespan‐extending drugs. Enriched terms include various functional categories related to glutathione and antioxidant activity, ion transport and metabolic processes. In addition, we found a modest but significant overlap between targets of lifespan‐extending drugs and known aging‐related genes, suggesting that some but not most aging‐related pathways have been targeted pharmacologically in longevity studies. DrugAge is freely available online for the scientific community and will be an important resource for biogerontologists.

Population aging is a major medical, social and economic challenge worldwide. Not surprisingly, identifying drugs and compounds that delay aging is of widespread importance (Moskalev *et al*., 2016), in particular, given recent advances in our understanding of the genetics of aging in model organisms (de Magalhaes *et al*., 2012). Indeed, dozens of studies have been performed in recent years in model organisms to assay compounds for longevity effects. The growing amount of data being generated increases the complexity of its analysis and the difficulty of placing findings in the context of previous studies. To help researchers study and prioritize drugs and compounds of relevance to aging, we developed DrugAge, a database of drugs and compounds that modulate aging. We also present our initial analyses and functional enrichment of DrugAge gene targets.

## The DrugAge database

DrugAge aims to provide high‐quality summary data on lifespan‐extending drugs and compounds in model organisms. Data were primarily assembled using literature searches (see Experimental Procedures in the Supporting information). Additionally, we used other databases and submissions from the scientific community, resulting in 324 research articles. DrugAge data focus solely on assays performed in standard conditions (Experimental Procedures in Supporting information). Only compounds, drugs and substances extending lifespan in a statistically significant manner in at least one experiment were included, but conflicting and negative results were then also added to those entries to provide a balanced literature survey.

**Figure 1:**
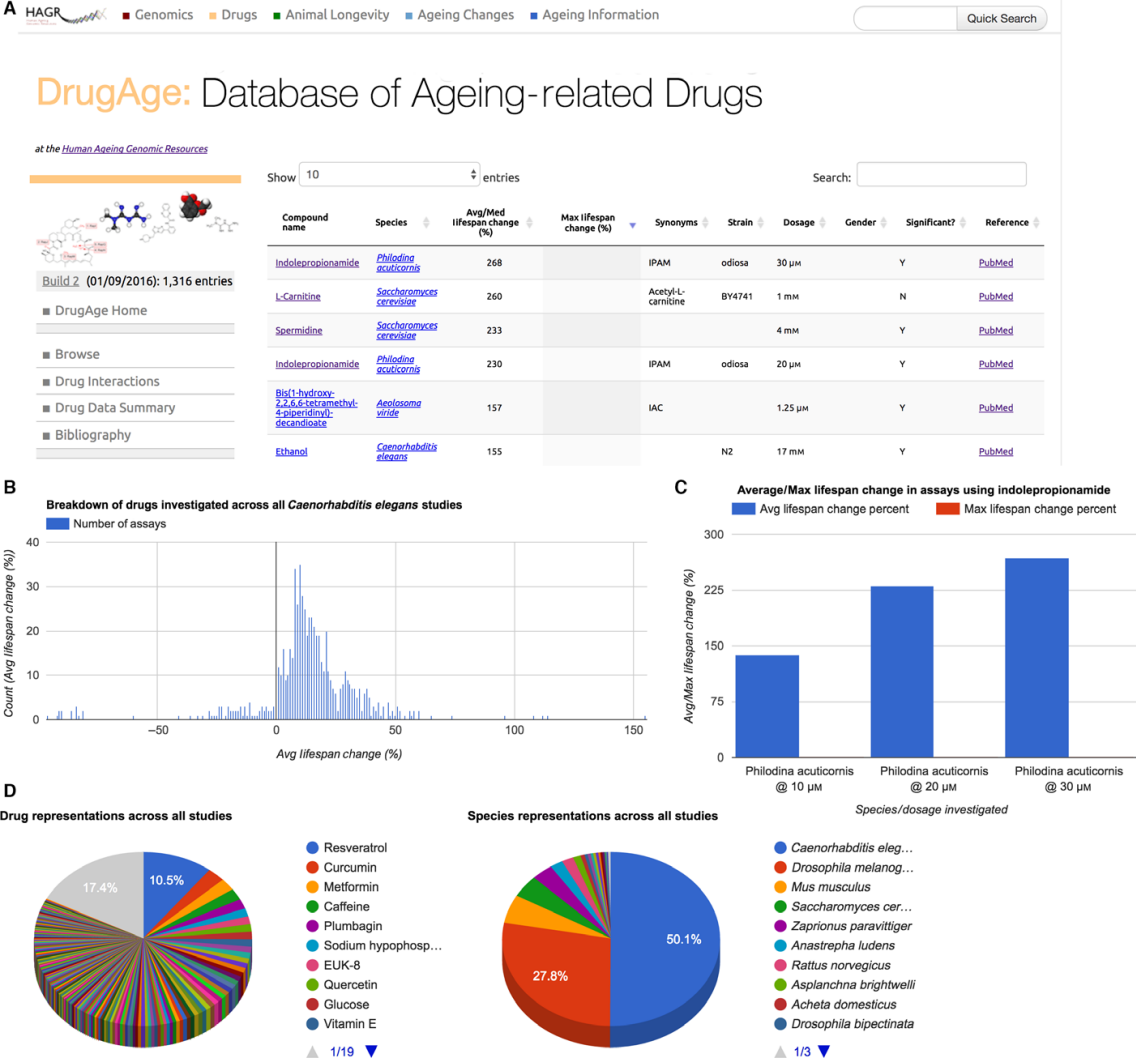
Several snapshots of DrugAge's Web interface. (A) Snapshot of the DrugAge browser, accompanied by Web interface examples showing (B) the distribution of drug lifespan effects in *C. elegans*; (C) average lifespan results for the drug ‘indolepropionamide’; and (D) an overall species summary breakdown across the database.

DrugAge is searchable based on fields such as the compound name, species or lifespan effect. The current version (build 2) of the database comprises 1316 lifespan assays using 418 distinct compounds on 27 unique model organisms (including 70 individual strains), in particular worms (481 entries), flies (365 entries), yeast (55 entries) and mice (70 entries). The data are presented as tables and interactive charts (Fig. 1). DrugAge is freely available online (http://genomics.senescence.info/drugs/), and data are also available for download. As we will continue to update DrugAge, we encourage feedback and further data submissions from the research community.

We developed a user‐friendly interface to search and retrieve information from DrugAge (Fig. 1). Key experimental parameters for each assay (average/median lifespan, maximum lifespan, strain, dosage and gender, if available), citations and a link to the original PubMed record are provided. The data browser also features drug and organism records, enabling detailed exploration of the aggregated results for a specific drug or species. Moreover, we found that 214 of the 418 compounds/substances have a known biological interaction in drug interactions databases (Experimental Procedures in Supporting information). As such, users can identify interacting genes and proteins in tabular form, and in network diagrams.

## DrugAge analyses and functional enrichment

**Figure 2:**
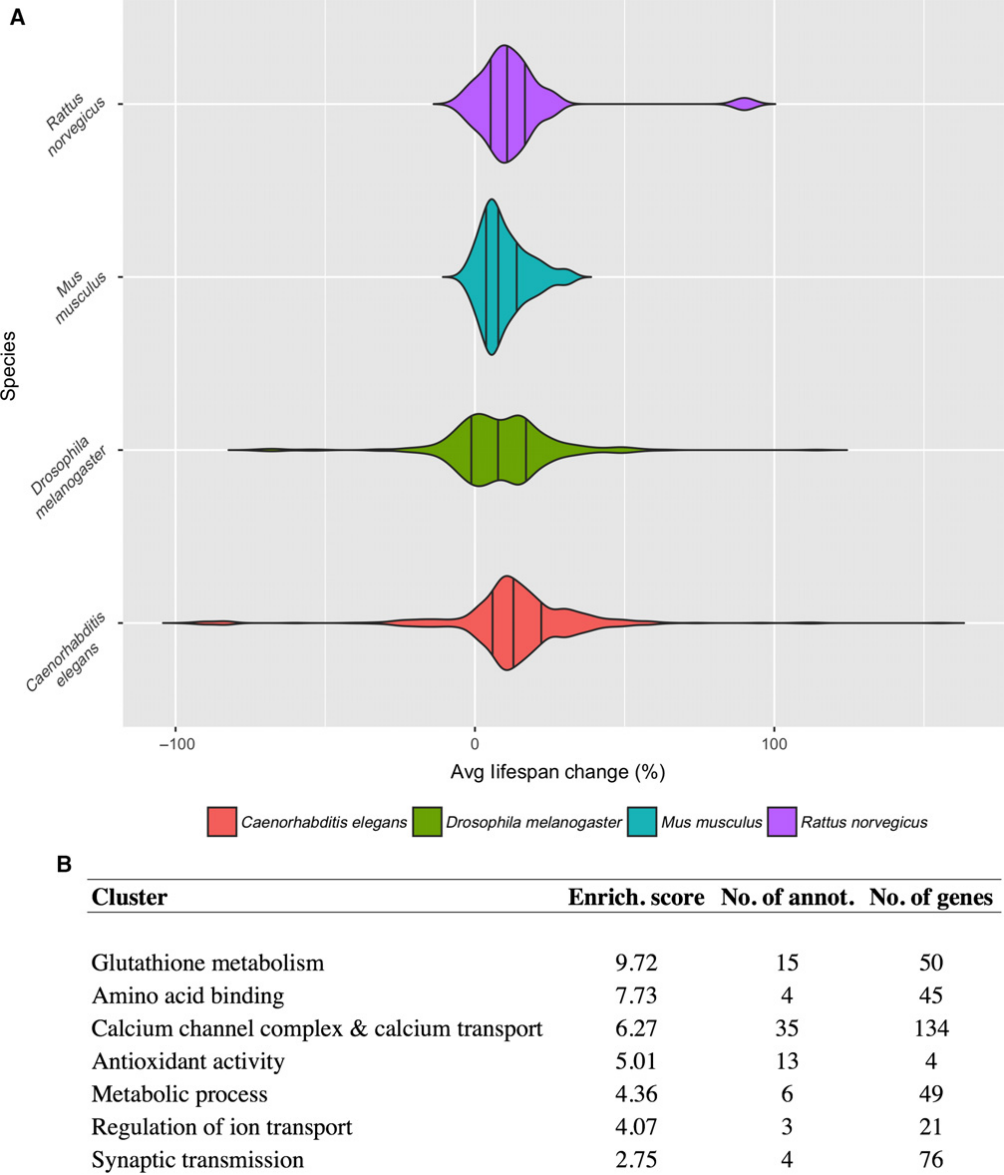
(A) Violin plot based on the average/median lifespan changes reported on 653 *C. elegans*, 357 *D. melanogaster*, 63 *M. musculus* and 23 *R. norvegicus* lifespan assays. (B) Top functional annotation clusters of gene targets of compounds in DrugAge. Only clusters with an enrichment score >4 are shown. Full results in Table S1.

We found that the magnitude of mean/median lifespan changes per assay is species‐dependent and effects are more modest in mice when compared to lower organisms (Fig. 2A). The extent of maximum lifespan changes is more modest and not as dissimilar across species (Fig. S1, Supporting information). We also noted a linear correlation (*r* ^2^ = 0.73) between average/median and maximum lifespan changes (Fig. S2, Supporting information). Lastly, we found a strong correlation between average/median (*r* ^2^ = 0.77) and maximum (*r* ^2^ = 0.81) lifespan changes in males and females, suggesting a modest sexual dimorphism (Fig. S3, Supporting information).

To analyse enriched processes in the targets of compounds in DrugAge, we used DGIdb (Wagner *et al*., 2016) to obtain a list of human gene interacting partners for all drugs in DrugAge (Experimental Procedures in Supporting information). Of 418 DrugAge compounds/substances, 90 were found to have a DGIdb record, resulting in a total of 411 distinct genes. Statistically significant enriched terms amongst gene targets of compounds in DrugAge include various functional categories related to glutathione and antioxidant activity, calcium and ion transport and metabolic processes (Fig. 2B for top results and Table S1, Supporting information for full results).

We explored the overlap between DrugAge interacting genes and genes previously associated with aging. This was carried out by obtaining the 1124 human orthologues of genes that extend lifespan in model organisms using the GenAge database (Tacutu *et al*., 2013). Of the 1124 genes, 287 (25.5%) were known to interact with drugs in DGIdb (Wagner *et al*., 2016). Of these, 65 (29.3%) overlap with DrugAge targets (Fig. S4, Supporting information), when 38 would be expected by chance. As such, we identified a modest but significant (*P*‐value < 0.01) degree of overlap between the genes arising from the two databases.

## Concluding remarks

DrugAge was designed to complement and expand existing databases in the context of aging. In particular, the Human Ageing Genomic Resources (HAGR), also developed by some of us, offers a collection of online databases for understanding aging in both humans and model organisms (Tacutu *et al*., 2013). HAGR focuses mostly on genes, and thus there is a need for a curated database of drugs and compounds that can extend lifespan in model organisms. Existing databases with lifespan‐extending drugs include AgeFactDB (http://agefactdb.jenage.de/) (Huhne *et al*., 2014), in turn based on data from HAGR and SAGEWEB (http://sageweb.org/), and Geroprotectors.org (http://geroprotectors.org/) (Moskalev *et al*., 2015). DrugAge incorporates data from all these resources and improves on them by providing a more extensive and systematic repertoire of lifespan‐extending drugs, compounds and substances. Indeed, we followed the high standards for manual curation, user‐friendly interface and performance of HAGR (Tacutu *et al*., 2013). Besides, DrugAge integrates drug–gene interactions and cross‐links to aging‐related genes, allowing a deeper examination of lifespan‐extending drugs. Interestingly, we have noted a sharp increase in the number of drug assays carried out yearly post‐2000 (Fig. S5, Supporting information), indicating the timeliness of DrugAge and of pharmacological interventions in aging.

Our initial analysis of DrugAge reveals various trends and insights. We observed that lifespan‐extending effects are more modest in mice than in lower organisms, as would be expected. In addition, we observed a strong correlation between average/median and maximum lifespan changes, as well as between lifespan‐extending effects in males and females. The significant but modest overlap between targets of lifespan‐extending drugs and known aging‐related genes suggests that some but not most aging‐related pathways have been targeted pharmacologically in longevity studies. Lastly, we explored functions and pathways enriched in the targets of lifespan‐extending drugs. The most relevant categories are related to glutathione and antioxidant activity, although a caveat to this analysis is that it does not take into account researcher biases in historically studying particular pathways and proteins (and the drugs targeting them) more than others.

In conclusion, we developed a new database of lifespan‐extending compounds and drugs in model organisms that reflect our current knowledge of pharmacological manipulations of aging. Given its scientific quality and rigour, DrugAge will be the benchmark in the field and will be of great value to researchers.

## Funding

This work was supported by a Wellcome Trust grant (104978/Z/14/Z) to J.P.M, and funding from the Israel Ministry of Science and Technology to A.B.

## Conflict of interest

None declared.

## Author contributions

JPM conceived and coordinated the project. DB, DT and JPM analysed the data. DB, DT, HT, MW, SS, SF, AA, MF, PM, TG, RC, EADS, AB, NA, JG, MP, VEF, AZ, AM and JPM contributed reagents/materials/analysis tools. DB, DT and JPM wrote the manuscript.

## Supporting information


**Fig. S1** Violin plot of the maximum lifespan changes obtained in 140 *C. elegans*, 74 *D. melanogaster* and 27 *M. musculus* lifespan assays.
**Fig. S2** Scatter plot of average known lifespan change (horizontal axis) and maximum lifespan change (vertical axis) from 356 assays that measured both.
**Fig. S3** Side‐by‐side display of the linear correlation between average (*r* = 0.88) and maximum lifespan (*r* = 0.90) changes among gender‐paired lifespan assays (*n* = 141) from 11 species.
**Fig. S4** Venn diagram displaying the number of unique and shared genes between DrugAge and human orthologues of GenAge lifespan‐extending genes.
**Fig. S5** Log scale plot displaying the distribution of average/median lifespan effects in DrugAge across publication years.

**Table S1** Functional annotation clusters of gene targets of compounds in DrugAge.
