# Supplementary material for: The DrugAge database of aging‐related drugs
Source: Aging Cell. 2017 Mar 16;16(3):594–7. doi: 10.1111/acel.12585 (PMC5418190; doi:10.1111/acel.12585)
Supplement: Supplementary file 1 — Fig. S1 Violin plot of the maximum lifespan changes obtained in 140 C. elegans, 74 D. melanogaster and 27 M. musculus lifespan assays. Fig. S2 Scatter plot of average known lifespan change (horizontal axis) and maximum lifespan change (vertical axis) from 356 assays that measured both. Fig. S3 Side‐by‐side display of the linear correlation between average (r = 0.88) and maximum lifespan (r = 0.90) changes among gender‐paired lifespan assays (n = 141) from 11 species. Fig. S4 Venn diagram displaying the number of unique and shared genes between DrugAge and human orthologues of GenAge lifespan‐extending genes. Fig. S5 Log scale plot displaying the distribution of average/median lifespan effects in DrugAge across publication years. [file ACEL-16-594-s001.pdf]

Supplementary Information

Barardo et al., The DrugAge Database of Ageing-Related Drugs

Experimental Procedures

Database curation

The literature mining for DrugAge came from three sources: data submitted by the scientific community; mining of pre-existing aging-related databases; and PubMed queries. The existing databases queried were: AgeFactDB[1]; Geroprotectors.org[2]; and the Aging Genes and Interventions Database[3].

To be a candidate for inclusion in DrugAge, a molecule or substance (e.g. whole apple extract) must extend lifespan (average, median or maximum), reaching statistical significance in at least one lifespan assay in one model organism. All literature mined was manually curated and subjected to quality-control in order to standardize compound naming conventions, species, and strains.

Exclusion of research papers or some of their assays (partial exclusion) were based on the following criteria: assays without a control group or statistical analysis of the results (exceptions were left to the curator’s discretion in order to include some research papers that included information deemed essential for DrugAge e.g. a large-scale assay); assays that used disease or short-lived strains; non-standard diets, e.g. high-fat diets, or environmental conditions, e.g. high-temperature; experiments that used mutant strains, e.g. strains with

specific genes knocked out; non-English papers; data from research papers not indexed by PubMed.

The above protocol for literature mining and data curation will continue to be followed as we update DrugAge regularly by releasing new builds in the future.

Database and user interface

DrugAge is built on a MySQL 5.5.48 relational database running on a Linux operating system. The user interface is provided through PHP 5.4.36 dynamic pages, served via Apache 2.2.29. The DrugAge UI also features various tables implemented through the DataTables JavaScript library, whilst the visualizations have been developed using the GoogleCharts API.

A total of 35,361 drug-gene interactions were downloaded using version 2.0 of the DGIdb, specifically the interactions.tsv file. Drug-protein interaction data was obtained by querying the STITCH (version 4.0) API for the drugs specific to DrugAge and with a medium confidence score above 0.450, resulting in 7495 stored drug-protein interactions. Of the 418 compounds and products in DrugAge, 90 have known human gene interactions in the DGIdb, and 187 were found to have a known human protein interaction in STITCH.

Analyses of DrugAge data

Of the 27 species currently present in DrugAge, only a limited number of them are analyzed because the rest of species were excluded due to insufficient

sample size. All figures with violin plots were graphed using RStudio[4] (version 0.99.879) running R[5] (version 3.2.3).

Enrichment analysis

Using data obtained from DGIdb 2.0, drug-gene interactions for DrugAge drugs were obtained by joining the DGIdb 'drug_primary_name' field to the DrugAge compound name field. In order to optimize the number of hits, we endeavoured to ensure the compound name field in DrugAge matched PubChem synonyms. From a possible 418 compounds/substances in DrugAge, 90 were found to have at least one DGIdb record, translating to 411 distinct interacting genes as input. Functional enrichment analysis using the Database of Annotation Visualization and Integrated Discovery (DAVID)[6] was performed to identify overrepresented categories. The analysis was done by running the Functional Annotation Clustering module under default parameters. The statistical enrichment analysis required a background dataset, which was taken as the 3090 distinct Ensembl Gene Ids found in DGIdb.

GenAge versus DrugAge gene overlap

The overlap between human genes mapped to DrugAge and in GenAge was evaluated by Blaker's Exact Test using the "exact2x2" R package[7]. The background chosen was the set of 3090 DGIdb genes. Genes counted as "DrugAge" in Figure S4 are all DrugAge-interacting genes in DGIdb. The subset of human orthologues of GenAge life-extending genes contained 1124 unique

human genes, but it was then reduced to the subset of 287 genes that are present in the DGIdb.

Bibliography

1. Hühne, R., Thalheim, T. & Sühnel, J. AgeFactDB - The JenAge Ageing Factor Database - Towards data integration in ageing research. *Nucleic Acids Res.* **42,** (2014).
2. Moskalev, A. *et al.* Geroprotectors.org: A new, structured and curated database of current therapeutic interventions in aging and age-related disease. *Aging (Albany. NY).* **7,** 616–628 (2015).
3. Kaeberlein, M., Jegalian, B. & McVey, M. AGEID: A database of aging genes and interventions. *Mechanisms of Ageing and Development* **123,** 1115–1119 (2002).
4. RStudio. RStudio: Integrated development environment for R. *The Journal of Wildlife Management* **75,** (2012).
5. R Developement Core Team. R: A Language and Environment for Statistical Computing. *R Foundation for Statistical Computing* **1,** 409 (2015).
6. Huang, D. W., Lempicki, R. a & Sherman, B. T. Systematic and integrative analysis of large gene lists using DAVID bioinformatics resources. *Nat. Protoc.* **4,** 44–57 (2009).
7. Fay, M. P. Confidence intervals that match fisher’s exact or blaker's exact tests. *Biostatistics* **11,** 373–374 (2010).

Supplementary Figures

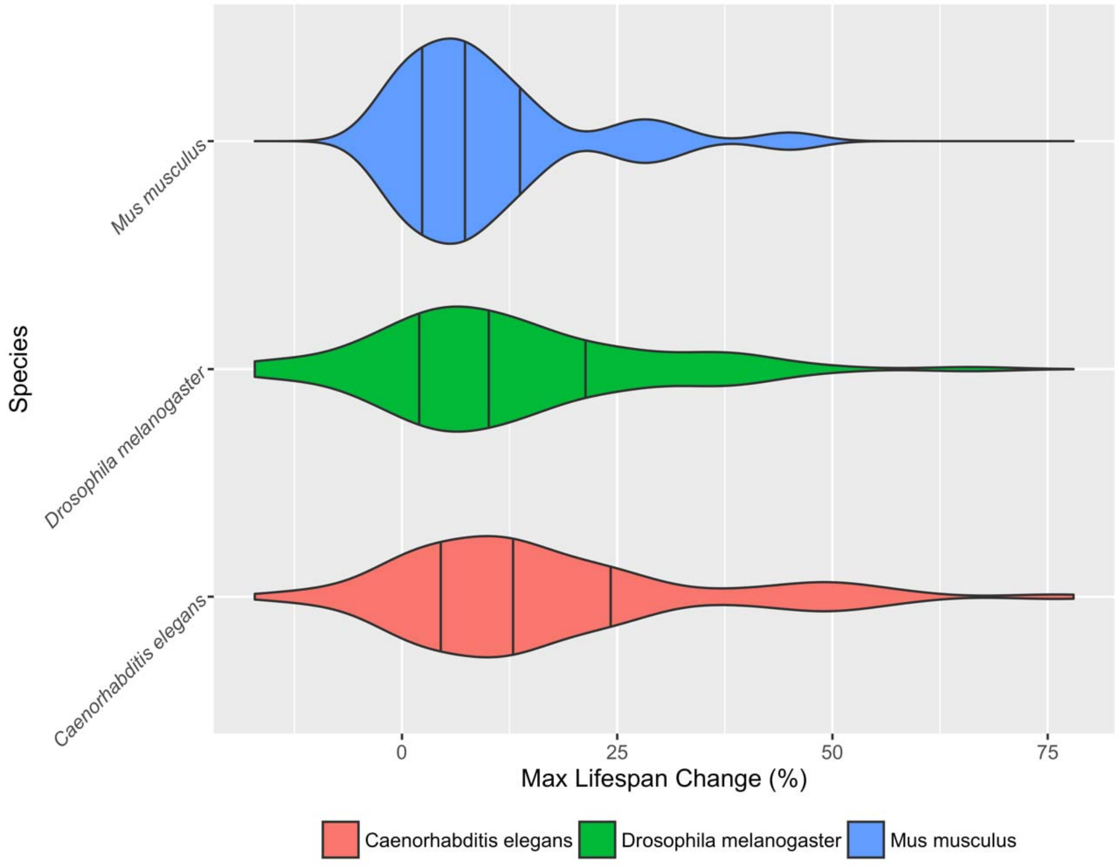

Figure S1. Violin plot of the maximum lifespan changes obtained in 140 *C. elegans*, 74 *D. melanogaster* and 27 *M. musculus* lifespan assays.

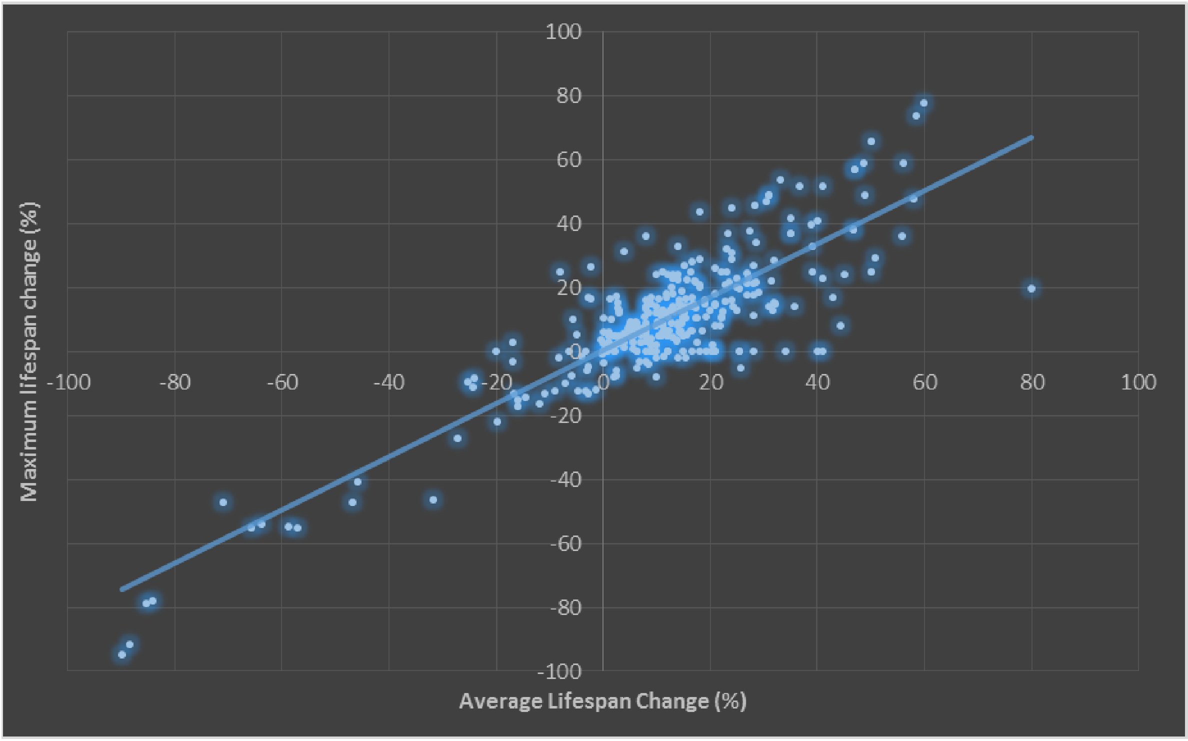

Figure S2. Scatter plot of average known lifespan change (horizontal axis) and maximum lifespan change (vertical axis) from 356 assays that measured both. Pearson correlation of 0.85.

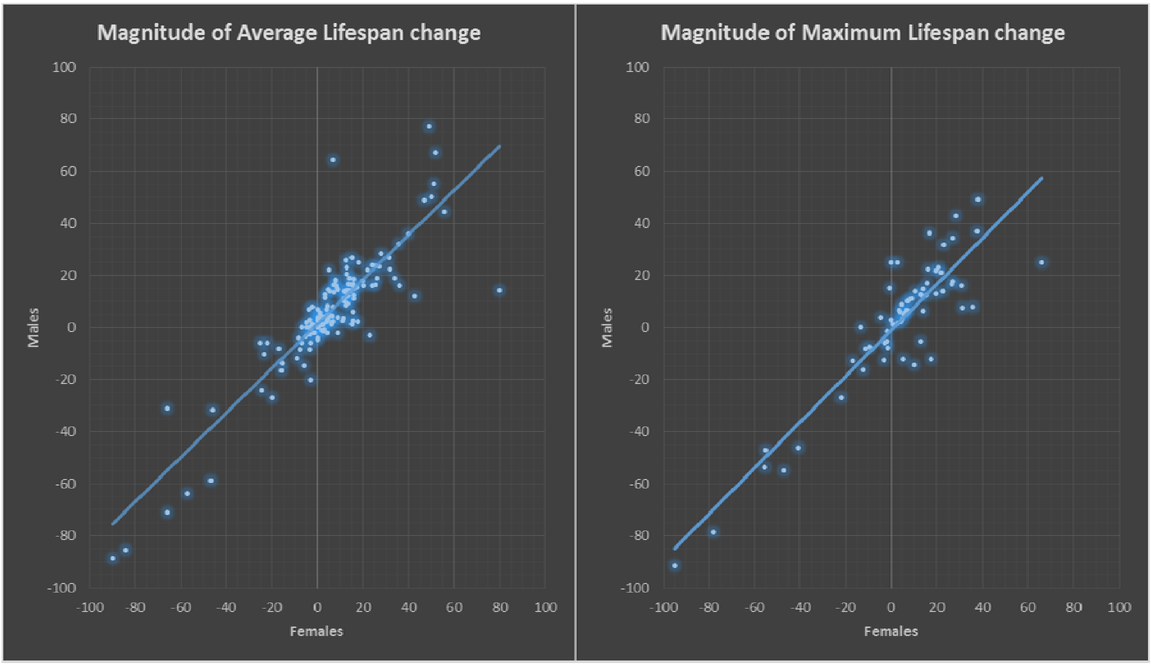

Figure S3. Side-by-side display of the linear correlation between average ($r = 0.88$) and maximum lifespan ($r = 0.90$) changes among gender-paired lifespan assays ($n = 141$) from 11 species.

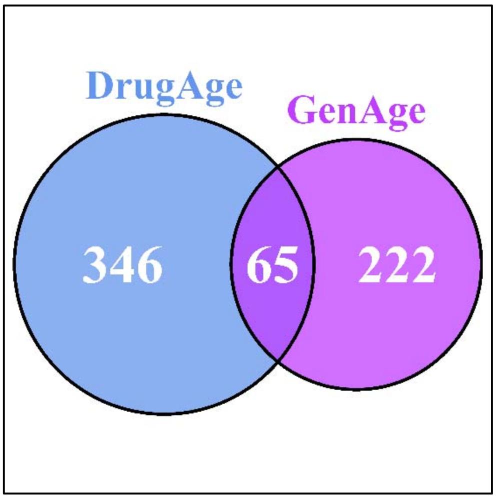

Figure S4. Venn diagram displaying the number of unique and shared genes between DrugAge and human orthologues of GenAge lifespan-extending genes. In total, 65 GenAge genes overlap with DrugAge targets when 38 would be expected by chance.

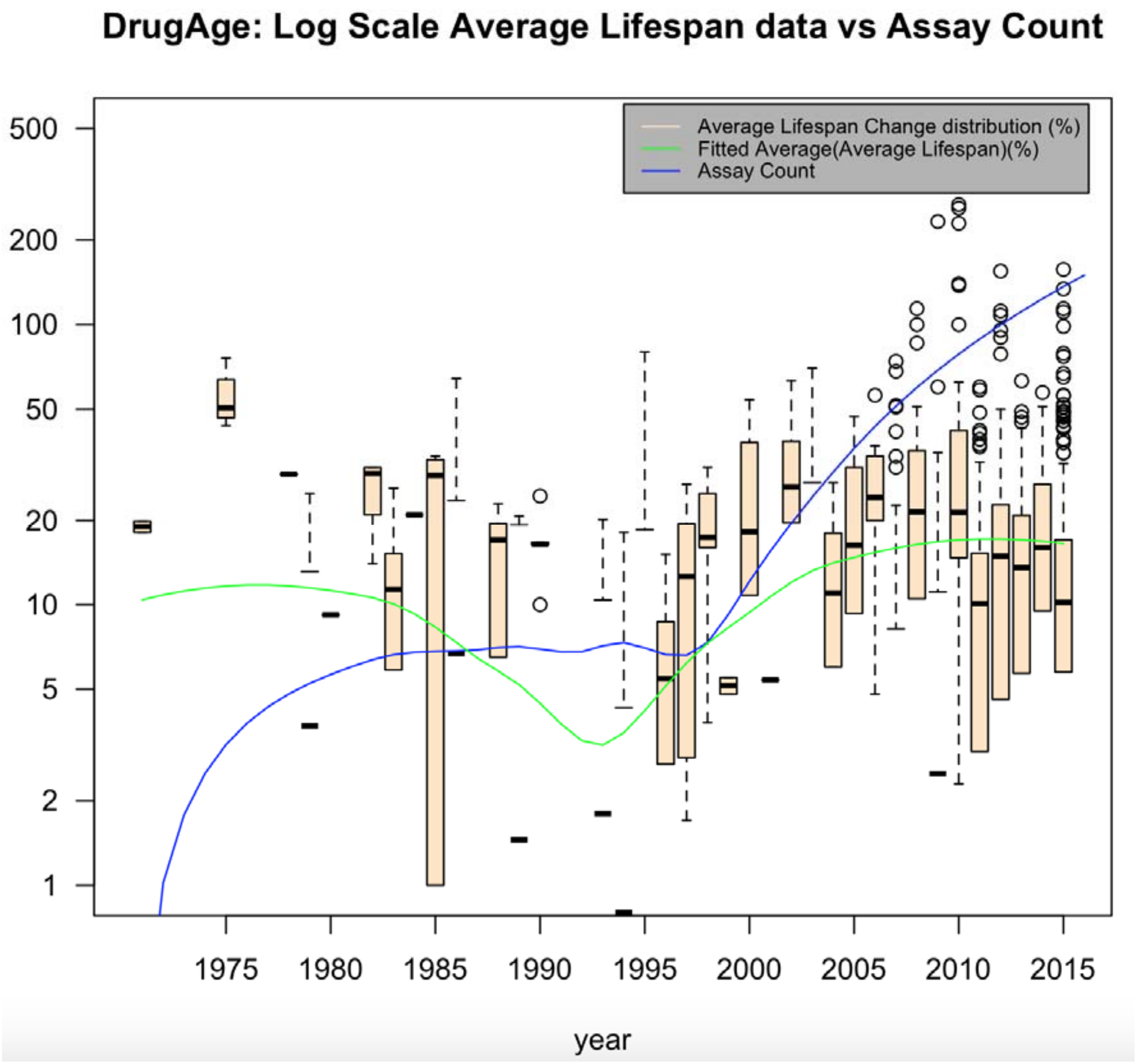

Figure S5. Log scale plot displaying the distribution of average/median lifespan effects in DrugAge across publication years. Overlaid are fitted representations (least squares) of the mean lifespan effect by year and the assay count by year.

Additional Files

TableS1.xlsx:

Table S1: Functional annotation clusters of gene targets of compounds in DrugAge.
